# Costs of clinical events in type 2 diabetes mellitus patients in the Netherlands: A systematic review

**DOI:** 10.1371/journal.pone.0221856

**Published:** 2019-09-06

**Authors:** Alexander V. van Schoonhoven, Judith J. Gout-Zwart, Marijke J. S. de Vries, Antoinette D. I. van Asselt, Evgeni Dvortsin, Pepijn Vemer, Job F. M. van Boven, Maarten J. Postma

**Affiliations:** 1 Unit of PharmacoTherapy, Epidemiology & Economics (PTE2), Department of Pharmacy, University of Groningen, Groningen, the Netherlands; 2 Asc Academics, Groningen, the Netherlands; 3 Department of Nephrology, University of Groningen, University Medical Centre Groningen (UMCG), Groningen, the Netherlands; 4 Department of Epidemiology, University Medical Centre Groningen, Groningen, the Netherlands; 5 Department of Health Sciences, University of Groningen, University Medical Centre Groningen (UMCG), Groningen, the Netherlands; 6 Department of General Practice & Elderly Care, University of Groningen, University Medical Center Groningen (UMCG), Groningen, the Netherlands; 7 Department of Clinical Pharmacy & Pharmacology, University of Groningen, University Medical Centre Groningen (UMCG), Groningen, the Netherlands; 8 Department of Economics, Econometrics & Finance, University of Groningen, Faculty of Economics & Business, Groningen, The Netherlands; Mahidol University, THAILAND

## Abstract

**Background:**

Type 2 diabetes mellitus (T2DM) is an established risk factor for cardiovascular and nephropathic events. In the Netherlands, prevalence of T2DM is expected to be as high as 8% by 2025. This will result in significant clinical and economic impact, highlighting the need for well-informed reimbursement decisions for new treatments. However, availability and consistent use of costing methodologies is limited.

**Objective:**

We aimed to systematically review recent costing data for T2DM-related cardiovascular and nephropathic events in the Netherlands.

**Methods:**

A systematic literature review in PubMed and Embase was conducted to identify available Dutch cost data for T2DM-related events, published in the last decade. Information extracted included costs, source, study population, and costing perspective. Finally, papers were evaluated using the Consolidated Health Economic Evaluation Reporting Standards (CHEERS).

**Results:**

Out of initially 570 papers, 36 agreed with the inclusion criteria. From these studies, 150 cost estimates for T2DM-related clinical events were identified. In total, 29 cost estimates were reported for myocardial infarction (range: €196-€27,038), 61 for stroke (€495-€54,678), fifteen for heart failure (€325-€16,561), 24 for renal failure (€2,438-€91,503), and seventeen for revascularisation (€3,000-€37,071). Only four estimates for transient ischaemic attack were available, ranging from €587 to €2,470. Adherence to CHEERS was generally high.

**Conclusions:**

The most expensive clinical events were related to renal failure, while TIA was the least expensive event. Generally, there was substantial variation in reported cost estimates for T2DM-related events. Costing of clinical events should be improved and preferably standardised, as accurate and consistent results in economic models are desired.

## Introduction

Type 2 diabetes mellitus (T2DM) is an established risk factor for vascular complications, cardiovascular events and renal failure [1,2]. Also, T2DM is the most prevalent chronic disease in the Netherlands. In 2014, an estimated 960,000 patients with T2DM were known to the general practitioner, which is about 5.7% of the Dutch population [3,4]. The prevalence is expected to be as high as 8% in the year 2025 [5]. Besides significant clinical impact, this will result in profound increases in healthcare expenditures and highlights the need for appropriate assessment of T2DM drugs’ cost-effectiveness and well-informed reimbursement decisions.

T2DM treatment is aiming to normalise blood sugar levels, blood pressure and lipids with the ultimate goal to prevent cardiovascular and renal complications. Major cardiovascular complications include myocardial infarction (MI), stroke, transient ischaemic attack (TIA), heart failure (HF), and revascularisation. Major renal complications include end-stage renal disease (ESRD), dialysis, and kidney transplantation. Given their significant impact on both patients’ health status as well as healthcare expenditures, a prerequisite for state-of-the-art health-economic evaluations is the full understanding and consistent use of T2DM-related complications’ costs, especially those of related cardiovascular and nephropathic events.

In the Netherlands, the National Health Care Institute (Zorginstituut Nederland, ZIN) provides guidelines for pharmacoeconomic research, highlighting key methodological issues that should be addressed for an adequate economic evaluation [6]. For instance, according to the guideline, economic evaluations should be carried out using a societal perspective, taking into account costs both inside and outside the healthcare system, and thus including e.g. productivity losses. Indeed, according to pharmacoeconomic guidelines, costs for clinical events are essential for designing adequate and valid health-economic models [6,7]. Yet, availability, a standardised measurement, and consistent use of costs of clinical events related to T2DM is limited. Here, we aim to systematically review available recent costing data for T2DM-related major cardiovascular and nephropathic events in the Netherlands.

## Methods

### Study design

A systematic literature review was conducted to identify all available publications specifying Dutch costs for clinical events commonly found in T2DM patients. This review was reported according to the Preferred Reporting Items for Systematic reviews and Meta-Analyses (PRISMA) literature review methodology [8], provided in S1 Table.

### Search strategy

The PubMed database was searched for publications between January 1^st^ 2005 and January 1^st^ 2018. An overview of keywords can be found in Table 1. In addition, reference lists of identified reviews and meta-analyses on the topic were searched for potentially relevant articles.

**Table 1:** Search terms used to identify studies reporting on Dutch type 2 diabetes mellitus clinical event costs.

Domain	Search terms
Subject	“costs and cost analysis” OR “cost-effectiveness” OR “cost-utility” OR “cost-benefit” OR “cost-effective” OR “economic evaluation” OR “economic analysis”
Events	“diabetes mellitus” OR “stroke” OR “myocardial infarction” OR “heart failure” OR “ischemic attack, transient” OR “myocardial revascularisation” OR “albuminuria” OR “acute renal injury” OR “renal insufficiency”
Setting	Netherlands
Date	2005/01/01-2018/01/01

Subsequently, Embase was also searched with adapted keywords from Table 1, corresponding with Emtree terms. In this analysis, results also found in MEDLINE were filterred out, as to improve efficiency.

### In- and exclusion criteria

Articles were included in this review if they met the following criteria:

The papers considered direct costs for the specified clinical events per patient in the Netherlands.

Papers required to be published between January 1^st^ 2005 and January 1^st^ 2018, to reflect recent data only, as older estimates may be “outdated” and irrelevant for present day.

The paper evaluated at least one of the six predefined major clinical events (MI, stroke, TIA, HF, renal failure, and revascularisation).

Papers included T2DM patients or patients with clinical events commonly associated with T2DM. It should be noted that these clinical events are not restricted to T2DM patients and can occur in patients without T2DM as well. Therefore, given the focus on the costs for these clinical events, the patients in the included studies were not always T2DM patients.

The full-text of the paper required to be accessible.

Articles reflecting guidelines or study protocols, meeting reports, or case reports were excluded.

### Extracted information

Direct medical costs, such as those for hospitalisation, medication and rehabilitation, were included. Additionally, and in line with the Dutch preferred societal perspective, indirect costs such as productivity losses were identified and included. If comparative studies were identified, the costing in the standard-of-care arm was chosen to be included, as it reflects standard of practice more accurately. Next to costs, data extracted included the cost source, study population, and the costing perspective (e.g. a healthcare payer, the hospital or the society).

### Analyses

Studies were categorised per clinical event, i.e., MI, stroke, TIA, HF, revascularisation, and renal failure. Subsequently, they were further sorted by year of costing, i.e. the price date, were reported. Cost estimates included the acute costs for the event, and, if available, cost for follow-up (monthly or annual, depending on availability). These analyses were of a descriptive nature, as only a limited number of cost estimates were trial-based, limiting generalisability.

To ease comparison, we also reported maximum and minimum annual costs in 2018 euros, with standard Dutch inflation rates used for standardising costs from previous years [9].

For papers that were health economic evaluations, adherence to the Consolidated Health Economic Evaluation Reporting Standards (CHEERS) was also assessed to put the individual cost estimates into perspective, regarding time horizon, reporting perspective, et cetera [10].

## Results

### Search results

**Fig 1:**
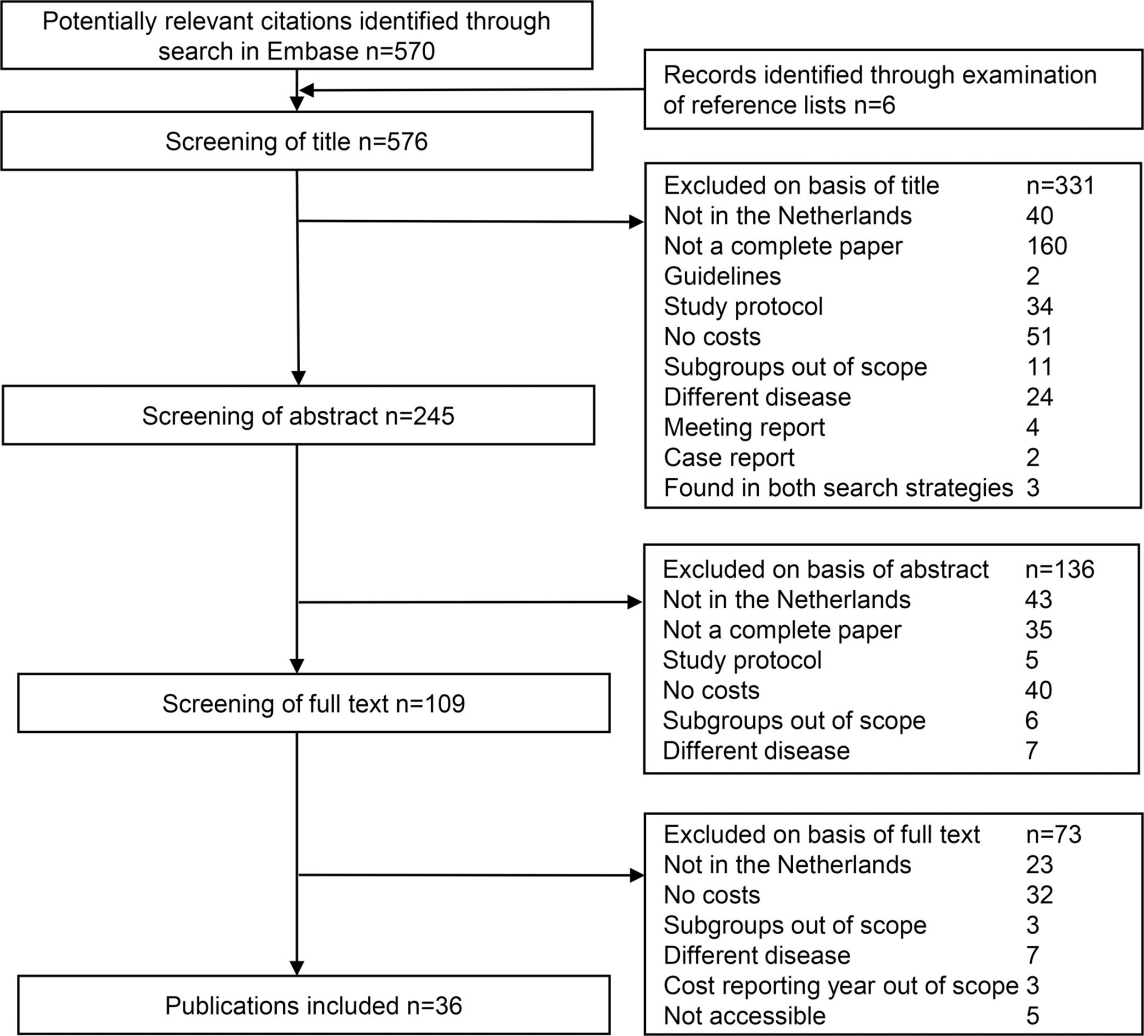
Flow chart of literature search.

The results of the systematic literature search are displayed in the flowchart in Fig 1.

### Overview

In total, 36 studies met our inclusion criteria. From these studies, 150 cost estimates for clinical events related to T2DM were identified. All papers provided the year of costing, except for three [11–13]. For the first two studies, the year of costing was assumed to be the year before their respective publication, while for the latter, the year of costing was found in one of its references.

Table 2 provides the characteristics of the included studies. Nineteen analyses took a healthcare payer perspective, seven a societal perspective, six a hospital perspective, one a third-party payer perspective, and three articles did not specify the perspective used. Of all studies, eight explicitly stated that in addition to direct costs, indirect costs were also taken into account [1,12,14–19]. Four of these studies took productivity losses into account, and these four all used a societal perspective [1,14,16,18]. The other four studies took overhead costs into account, although these are not considered indirect costs in health economics.

**Table 2:** Characteristics of included studies.

	Study	Type of study	Patients	Intervention assessed	Clinical events costs included	Cost perspective
1	Adarkwah et al. 2011 [32]	Modelling	Patients newly diagnosed with T2DM	ACE-inhibitor	Renal failure	Healthcare payer
2	Anastasiadis et al. 2013 [11]	Modelling	Patients undergoing CABG	Extracorporeal circulation	Revascularisation	Healthcare payer
3	Baeten et al. 2010 [26]	Modelling	Hospitalised stroke patients	Stroke services	Stroke	Healthcare payer
4	Boersma et al. 2006 [33]	Modelling	Patients with chronic heart failure	Valsartan	- MI - Stroke - HF - Revascularisation	Healthcare
5	Boersma et al. 2010 [34]	Modelling	Patients with elevated albuminuria levels	Various population-based screen-and-treat scenarios for elevated albuminuria levels	- CV death - Renal failure	Healthcare payer
6	Boyne et al. 2013 [35]	Modelling	Patients with heart failure	Telemonitoring analysis	HF	Healthcare payer
7	Buisman et al. 2015 [20]	Modelling	Patients with recent ischaemic stroke or TIA	n.a.	- Stroke - TIA	Healthcare payer
8	De Vries et al. 2014 [36]	Modelling	Patients newly diagnosed with T2DM	Statins	- MI - Stroke	Healthcare payer
9	Greving et al. 2011 [28]	Modelling	Healthy men and women aged 45–75 years	Statins	- MI - Stroke	Healthcare payer
10	Heeg et al. 2007 [37]	Modelling	Patients receiving PCI	Long term clopidogrel	- MI - Stroke - Revascularisation	Healthcare payer
11	Heyde et al. 2007 [12]	Trial	Patients receiving PCI	Short-term observation after procedure	Revascularisation	Hospital
12	Hofmeijer et al. 2013 [21]	Modelling	Stroke patients aged 60 years or younger	Surgical Decompression	Stroke	Healthcare payer
13	Hunt et al. 2017 [24]	Modelling	Patients with T2DM uncontrolled on basal insulin	Insulin degludec/liraglutide	- MI - Stroke - HF -Renal failure	Healthcare payer
14	Jacobs et al. 2018 [38]	Modelling	Patients 65 years and over receiving seasonal influenza vaccination	Screening for AF in primary care with MyDiagnostick	- MI - Stroke	Societal
15	Kauf et al. 2005 [30]	Modelling	Patients treated in hospital for acute MI	n.a.	- MI - Revascularisation	Hospital
16	Mazairac et al. 2013 [14]	Modelling	Patients with ESRD	Hemodiafiltration	Renal failure	Societal
17	Nathoe et al. 2005 [39]	Trial	Off-pump coronary artery bypass		- MI - Stroke - Revascularisation	Not specified
18	Osnabrugge et al. 2015 [40]	Modelling	Patients with three-vessel or left main CAD	PCI vs. bypass surgery	Revascularisation	Healthcare
19	Peltola et al. 2013 [22]	-	Stroke patients	n.a.	Stroke	Hospital
20	Ramos et al. 2017 [41]	Modelling	Patients with chronic heart failure and reduced ejection fraction	Sacubitril/valsartan	- MI - TIA - HF - Renal failure - Revascularisation	Societal
21	Roze et al. 2016 [29]	Modelling	Patients with T2DM uncontrolled on insulin multiple day injections	CSII	- MI - Stroke - HF - Renal failure	Third-party payer
22	Soekhlal et al. 2013 [23]	Costing	Patients hospitalised for acute MI	n.a.	MI	Not specified
23	Stevanović et al. 2014 [42]	Modelling	Patients with non-valvular AF	Apixaban	- MI - Stroke	Healthcare payer
24	Struijs et al. 2006 [27]	Modelling	Stroke patients	n.a.	Stroke	Not specified
25	Tan et al. 2009 [15]	Costing	n.a.	n.a.	- MI - Stroke	Hospital
26	Tholen et al. 2010 [16]	Modelling	Patients with recent TIA or minor ischaemic stroke	CT angiography	Stroke	Societal
27	Tiemann 2008 [17]	Modelling	Healthy males between 50 and 60	n.a.	MI	Hospital
28	Vaidya et al. 2014 [43]	Modelling	Suspected cardiac chest pain patients	several	MI	Healthcare payer
29	Van Eeden et al. 2015 [18]	Trial	Patients post-stroke	n.a.	Stroke	Societal
30	Van Exel et al. 2005 [13]	Trial	Stroke patients	Stroke services	Stroke	Healthcare payer
31	Van Genugten et al. 2005 [44]	Trial	Patients with acute MI HF and LVSD	Eplerenone	HF	Societal
32	Van Giessen et al. 2016 [45]	Modelling	Patients with T2DM aged 60 years and over	Screening strategies to detect HF in T2DM patients	HF	Healthcare
33	Van Haalen et al. 2014 [1]	Modelling	Patients with T2DM receiving insulin	Dapagliflozin	- MI - Congestive HF - Stroke - Renal failure	Societal
34	Van Mastrigt et al. 2006 [19]	Trial	Low-risk CABG patients	Short-stay IC (8h of IC treatment)	Revascularisation	Hospital
35	Vemer et al. 2010 [46]	Modelling	Smoking individuals	Smoking cessation	Stroke	Healthcare payer
36	Verhoef et al. 2014 [47]	Modelling	Patients with AF, age 70, initiating oral anticoagulant therapy	Apixaban, rivaroxaban, dabigatran	- MI - Stroke -TIA	Healthcare payer

*ACE* angiotensin-converting enzyme, *AF* atrial fibrillation, *CABG* coronary artery bypass grafting, *CAD* coronary artery disease, *CSII* continuous subcutaneous insulin infusion, *CV* cardiovascular, *CT* computed tomographic, *HF* heart failure, *IC* intensive care, *LVSD* left ventricular systolic dysfunction, *MDI* multiple daily injections, *MI* myocardial infarction, *n*.*a*. not applicable, *PCI* percutaneous coronary intervention, *T2DM* type 2 diabetes mellitus, *TIA* transient ischaemic attack

Five studies used the Diagnosis Treatment Combination (Diagnose Behandel Combinatie, DBC), the Dutch case-mix categorisation aligned with resource use and applied for reimbursement of hospitals [20–24]. These DBCs are comparable with diagnosis related group (DRG) based systems used in other countries, although certain differences do exist, such as goal and scope [25]. In three studies, information on resource use was gathered from the EDISSE trial [13,26,27], while the sources for the other papers concerned registries, case record files, cost diaries, trials, billing systems, or cost estimates from older costing studies.

The Dutch Manual for Costing in Economic Evaluations was referenced by eighteen studies (62%), eleven of which used the manual to derive standard prices. Tariffs provided by the Dutch Healthcare Authority (Nederlandse Zorgautoriteit, NZa) were used in four studies [20,23,28,29]. Furthermore, nine articles gathered unit costs from hospitals directly. Fourteen studies used at least one costing study to derive their cost estimates from. Of these fourteen studies, it was found that nine referenced at least one paper published before 2005, and four references reported their costs in Dutch guilders. The cost estimates derived from one paper were reported in 2002 US dollars [30]. These estimates were converted to 2002 euros, using an exchange rate of 1 EUR = 0.95 USD [31].

### Costs for clinical events related to T2DM

**Fig 2:**
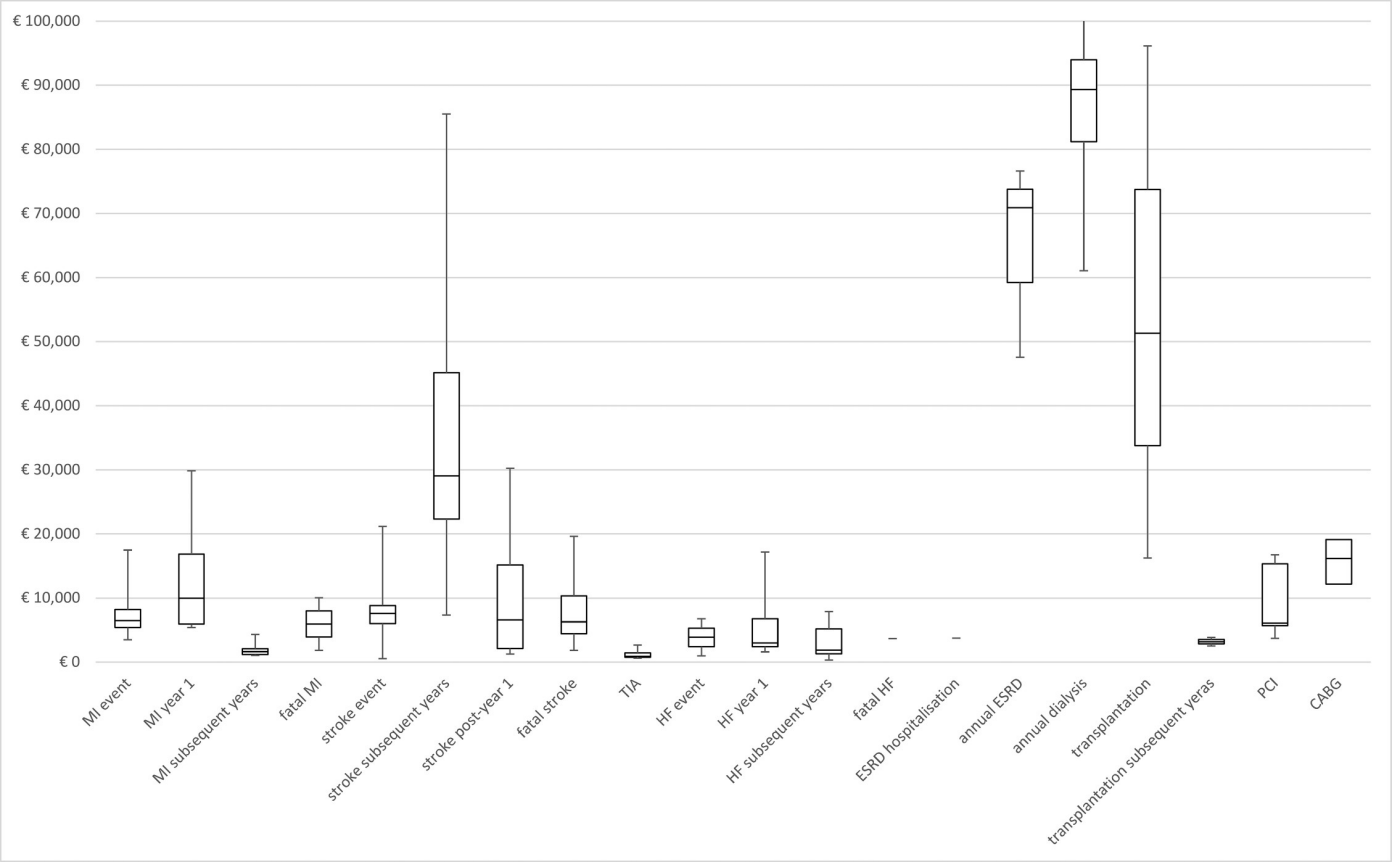
Costs for T2DM-related events in 2018 euros. *CABG* coronary arterial bypass grafting, *ESRD* end-stage renal disease, *HF* heart failure, *MI* myocardial infarction, *PCI* percutaneous coronary intervention, *TIA* transient ischaemic attack, *T2DM* type 2 diabetes mellitus.

Considerable variation among the reported costs was found. Fig 2 shows the minimum and maximum costs per clinical event, represented in 2018 EUR. Cost details for each clinical event are specified in sections 3.3.1 to 3.3.6, and overviews are provided in Tables 3–8.

**Table 3:** Summary of studies reporting costs for myocardial infarction.

Study	Specific type	Initial		Follow-up		Costs covered	
		Unit cost year 1 in € (year)	Source(s)	Unit cost year 2+ in € (year)	Source(s)	Direct costs	Indirect costs
Hunt et al. [24]	Acute MI	€6,341 (2015)	[23]	€1,026 (2015)	[23]	✓	
Ramos et al. [41]	Acute MI	€3,390 (2015)	Not specified			✓	✓
Jacobs et al. [38]	Acute MI	€5,021^a^ (2014)	[23]			✓	
	Post MI	€280^a^ (2014)	[23]			✓	
Roze et al. [29]	Acute MI	€5,138 (2013)	[36]	€1,932 (2013)	[36]	✓	
Stevanović et al. [42]	Acute MI	€5,021 (2013)	[23]			✓	
	Monthly maintenance	€196 (2013)	[28]			✓	
De Vries et al. [36]	Acute MI	€5,012 (2012)	[23]	€1,885 (2012)	[48]	✓	
Soekhlal et al. [23]	Acute MI	€5,021 (2012)	[17,49], DMC, tariffs			✓	
Vaidya et al. [43]	Acute MI	€12,446 (2012)	[50]	€2,092 (2012)	[50]	✓	
Verhoef et al. [47]	Acute MI	€5,021 (2012)	[28]			✓	
Van Haalen et al. [1]	MI	€27,038 (2011)	[28,51]	€1,132^b^ (2011)	[28]	✓	✓
	Fatal MI	€9,094 (2011)	Assumption			✓	
Greving et al. [28]	Acute MI	€17,342 (2008)	[52,53]	€1,054 (2008)	[52,53]	✓	
Tan et al. [15]	Acute MI	€5,338 (2005)	Hospital			✓	✓
Tiemann [17]	Acute MI	€5,599 (2005)	Hospital			✓	✓
Heeg et al. [37]	First 6 months	€10,250 (2004)	[52,54]	€1,750 (2004)	[52,54]	✓	
	Second 6 months	€2,500 (2004)	[52,54]			✓	
	Fatal MI	€1,500 (2004)	[52,54]			✓	
Kauf et al. [30]	Acute MI	€7,128^c^ (2002)	Analysts			✓	
Boersma et al. [33]	Acute MI	€5,823 (1999)	iMTA			✓	
Nathoe et al. [39]	MI	€12,395 (1999)	[55]			✓	

*CV* cardiovascular, *DMC* Dutch manual of costing, *iMTA* Institute for Medical Technology Assessment, *MI* myocardial infarction

^a^ Costs reported per 3-month cycles

^b^ No indirect costs applied to follow-up costs, since friction cost method was used for indirect costs

^c^ Study reported cost estimates in 2002 USD, converted to 2002 EUR for presentation in the table

**Table 4:** Summary of studies reporting costs for stroke.

Study	Specific type	Initial		Follow-up		Costs covered	
		Unit cost year 1 in € (year)	Source(s)	Unit cost year 2+ in € (year)	Source(s)	Direct costs	Indirect costs
Hunt et al. [24]	Stroke	€24,142 (2015)	[20]	€1,968 (2015)	[20]	✓	
	Fatal stroke	€5,523 (2015)	[20]			✓	
Jacobs et al. [38]	Acute minor IS	€19,146^a^ (2014)	[26]			✓	
	Post minor IS	€1,484^a^ (2014)	[26]			✓	
	Acute major IS	€44,138^a^ (2014)	[26]			✓	
	Post major IS	€3,958^a^ (2014)	[26]			✓	
	Fatal IS	€11,178^a^ (2014)	[56]			✓	
	Acute HS	€24,292^a^ (2014)	[26]			✓	
	Post HS	€1,691^a^ (2014)	[26]			✓	
	Fatal HS	€6,037^a^ (2014)	[56]			✓	
Roze et al. [29]	Stroke	€13,819 (2013)	[36]	€1,932 (2013)	[36]	✓	
	Fatal stroke	€8,603 (2013)	[36], tariffs			✓	
Stevanović et al. [42]	Mild stroke, first 6 months	€16,097 (2013)	[26]	€1,174^b^ (2013)	[26]	✓	
	Mild stroke, second 6 months	€4,470 (2013)	[26]			✓	
	Moderate stroke, first 6 months	€44,640 (2013)	[26]	€8,749^b^ (2013)	[26]	✓	
	Moderate stroke, second 6 months	€21,146 (2013)	[26]			✓	
	Severe stroke, first 6 months	€54,678 (2013)	[26]	€11,178^b^ (2013)	[26]	✓	
	Severe stroke, second 6 months	€26,711 (2013)	[26]			✓	
	Fatal stroke	€2,988 (2013)	[28]				
Buisman et al. [20]	IS, inpatient	€5,328 (2012)	DMC, DBC, tariffs			✓	
	IS, outpatient	€495 (2012)	DMC, DBC, tariffs			✓	
De Vries et al. [36]	Stroke	€13,480 (2012)	*Not accessible*	€1,885 (2012)	[48]	✓	
Van Eeden et al. [18]	Stroke, first 6 months	€21,731 (2012)	Bottom-up costing, DMC			✓	✓
	Stroke, second 6 months	€7,711 (2012)	Bottom-up costing, DMC			✓	✓
Verhoef et al. [47]	IS	€19,652 (2012)	[27]				
Van Haalen et al. [1]	Stroke	€45,430 (2011)	[26,57]	€4,497^c^ (2011)	[26]	✓	✓
	Fatal Stroke	€17,799 (2011)	Assumption			✓	
Hofmeijer et al. [21]	Stroke, first 3 years	€16,800 (2009)	Case record files, DMC, DRG			✓	
Boersma et al. [34]	CV event	€7,047 (2008)	[56]			✓	
	Fatal CV event	€1,593 (2008)	[56]			✓	
Greving et al. [28]	Major stroke	€36,173 (2008)	[53]	€21,122 (2008)	[53]	✓	
	Minor stroke	€6,343 (2008)	[53]	€1,085 (2008)	[53]	✓	
Peltola et al. [22]	Stroke	€5,262 (2008)	DBC			✓	
Tholen et al. [16]	Major IS	€43,650 (2007)	[58]	€25,487 (2007)	[58]	✓	
	Minor IS	€7,654 (2007)	[58]	€1,310 (2007)	[58]	✓	
Vemer et al. [46]	Stroke	€23,119 (2006)	[27]	€5,229 (2006)	[27]	✓	
Tan et al. [15]	Stroke	€6,264 (2005)	Bottom-up costing, hospitals			✓	✓
Heeg et al. [37]	Stroke, first 6 months	€17,750 (2004)	[59]	€4,500 (2004)	[59]	✓	
	Stroke, second 6 months	€6,750 (2004)	[59]			✓	
	Fatal Stroke	€3,250 (2004)	[59]			✓	
Baeten et al. [26]	Stroke, first 6 months	€24,837 (2003)	[60], DMC	€4,173^b^ (2003)	*Not accessible*	✓	
	Stroke, second 6 months	€9,826 (2003)	[60], DMC			✓	
Struijs et al. [27]	Stroke	€21,948^b^ (2000)	[55,60]	€4,993^d^ (2000)	[55,60]	✓	
Boersma et al. [33]	Stroke	€5,404 (1999)	iMTA			✓	
Nathoe et al. [39]	Stroke	€7,748 (1999)	[55]			✓	
Van Exel et al. [13]	Stroke, first 6 months	€16,000 (1999)	[60]			✓	

*DBC* diagnosis treatment combination, *DMC* Dutch manual of costing, *HS* haemorrhagic stroke, *iMTA* Institute for Medical Technology Assessment, *IS* ischaemic stroke

^a^ Costs reported per 3-month cycles

^b^ This cost estimate is a weighted mean calculated using the ratio between sexes as reported in the paper.

^c^ No indirect costs applied to follow-up costs, since friction cost method was used for indirect costs

^d^ Multiple cost estimates were reported, specified for gender and age, these values were based on women between the age of 75 and 84.

**Table 5:** Summary of studies reporting costs for TIA.

Study	Specific type	Initial		Follow-up		Costs covered	
		Unit cost year 1 in € (year)	Source(s)	Unit cost year 2+ in € (year)	Source(s)	Direct costs	Indirect costs
Ramos et al. [41]	TIA	€807 (2015)	Not specified			✓	✓
Buisman et al. [20]	TIA, inpatient	€2,470 (2012)	DMC, DRG			✓	
	TIA, outpatient	€587 (2012)	DMC, DRG			✓	
Verhoef et al. [47]	TIA	€949 (2012)	[61]			✓	

*DMC* Dutch manual of costing, *DRG* diagnosis related group, *TIA* transient ischaemic attack

**Table 6:** Summary of studies reporting costs for heart failure.

Study	Specific type	Initial		Follow-up		Costs covered	
		Unit cost year 1 in € (year)	Source(s)	Unit cost year 2+ in € (year)	Source(s)	Direct costs	Indirect costs
Hunt et al. [24]	Congestive HF	€5,479 (2015)	[62]	€954 (2015)	[62]	✓	
Ramos et al. [41]	HF	€945 (2015)	Not specified			✓	✓
Roze et al. [29]	Congestive HF	€2,870 (2013)	Tariffs	€325 (2013)	Tariffs	✓	
Van Haalen et al. [1]	Congestive HF	€15,571 (2011)	[63,64]	€6,762^a^ (2011)	Assumption	✓	✓
	Fatal congestive HF	€3,349 (2011)	Assumption			✓	
Van Giessen et al. [45]	NYHA I	€1,459^b^ (2011)	[65]			✓	
	NYHA II	€1,721^b^ (2011)	[65]			✓	
	NYHA III	€2,650^b^ (2011)	[65]			✓	
	NYHA IV	€7,156^b^ (2011)	[65]			✓	
Boyne et al. [35]	HF	€16,561 (2008)	DMC, hospital			✓	
Van Genugten et al. [44]	HF post-MI	€5,232 (2003)	Actual costs			✓	
Boersma et al. [33]	HF	€4,795 (1999)	iMTA			✓	

*DMC* Dutch manual of costing, *HF* heart failure, *iMTA* Institute for Medical Technology Assessment, *MI* myocardial infarction, *NYHA* New York Heart Association

^a^ No indirect costs applied to follow-up costs, since friction cost method was used for indirect costs

^b^ This cost estimate is a mean of detected and undetected values, for both men and women

**Table 7:** Summary of studies reporting costs for renal failure.

Study	Specific type	Initial		Follow-up		Costs covered	
		Unit cost year 1 in € (year)	Source(s)	Unit cost year 2+ in € (year)	Source(s)	Direct costs	Indirect costs
Hunt et al. [24]	HD	€81,256 (2015)	DBC	€81,256 (2015)	DBC	✓	
	PD	€88,749 (2015)	DBC	€88,749 (2015)	DBC	✓	
	Renal transplantation	€49,602 (2015)	[66]	€2,438 (2015)	[66]	✓	
Ramos et al. [41]	ESRD hospitalisation	€3,640 (2015)	Not specified			✓	✓
Roze et al. [29]	HD	€89,447 (2013)	Tariffs	€89,447 (2013)	Tariffs	✓	
	PD	€66,434 (2013)	Tariffs	€66,434 (2013)	Tariffs	✓	
	Renal transplantation	€91,503 (2013)	Tariffs	€3,680 (2013)	Tariffs	✓	
Van Haalen et al. [1]	ESRD	€69,440 (2011)	[67–69]	€64,251^a^ (2011)	[67]	✓	✓
Adarkwah et al. [32]	ESRD	€42,110 (2010)	[70]			✓	
	Renal transplantation	€14,387 (2010)	[70]			✓	
	Dialysis	€79,112 (2010)	[70]			✓	
	Home/in-centre HD	€83,217 (2010)	[70]			✓	
	CAPD	€54,067 (2010)	[70]			✓	
	CCPD	€69,546 (2010)	[70]			✓	
Mazairac et al. [14]	HD	€86,086 (2009)	[71,72], DMC, hospital			✓	✓
	HDF	€88,622 (2009)	[71,72], DMC, hospital			✓	✓
Boersma et al. [34]	Dialysis	€72,460 (2008)	[73], DMC			✓	

*CAPD* continuous ambulatory peritoneal dialysis, *CCPD* continuous cycling peritoneal dialysis, *DBC* diagnosis treatment combination, *DMC* Dutch manual of costing, *ESRD* end-stage renal disease, *HD* haemodialysis, *HDF* haemodiafiltration, *PD* peritoneal dialysis

^a^ no indirect costs applied to follow-up costs, since friction cost method was used for indirect costs

**Table 8:** Costs for revascularisation.

Study	Specified type	Initial		Follow-up		Costs covered	
		Unit cost year 1 in € (year)	Source(s)	Unit cost year 2+ in € (year)	Source(s)	Direct costs	Indirect costs
Ramos et al. [41]	PCI	€5,951 (2015)	Not specified			✓	✓
	CABG	€11,304 (2015)	Not specified			✓	✓
Osnabrugge et al. [40]	PCI	€14,037 (2012)	Not specified			✓	
	CABG	€17,506 (2012)	Not specified			✓	
Anastasiadis et al. [11]	CABG with CECC	€18,010 (2012)^a^	Not specified			✓	
Heyde et al. [12]	PCI same-day discharge	€4,675 (2006)^a^	Hospital, DMC			✓	✓
	PCI overnight-stay	€4,933 (2006)^a^	Hospital, DMC			✓	✓
Heeg et al. [37]	PCI	€3,000 (2004)	[52,54]			✓	
	CABG	€10,250 (2004)	[52,54]			✓	
Kauf et al. [30]	PCI without stent	€12,528 (2002)^b^	Analysts			✓	
	PCI with stent	€13,076 (2002)^b^	Analysts			✓	
	CABG with CC	€37,071 (2002)^b^	Analysts			✓	
Van Mastrigt et al. [19]	CABG	€5,441 (2001)	DMC, hospital, questionnaires			✓	✓
Boersma et al. [33]	PCI with stent	€4,208 (1999)	iMTA			✓	
	PCI without stent	€3,511 (1999)	iMTA			✓	
Nathoe et al. [39]	PCI	€4,250 (1999)	[55]			✓	
	CABG	€11,472 (1999)	[55]			✓	

*CABG* coronary arterial bypass grafting, *CC* coronary catheterisation, *CECC* conventional extracorporeal circulation, *DMC* Dutch manual of costing, *iMTA* Institute for Medical Technology Assessment, *PCI* percutaneous coronary intervention

^a^ No year of costing available, assumed to be the year before publication

^b^ Study reported cost estimates in 2002 USD, converted to 2002 EUR for presentation in the table

### Myocardial infarction

In seventeen studies, 29 different cost estimates for MI were used, with costs calculated between 1999 and 2015 (Table 3). The papers used various methods to derive cost estimates, such as hospital database analyses, and expert opinions. It was possible to make a differentiation between first-year (acute) and follow-up costs. Seven studies also considered the follow-up costs after year one [1,24,28,29,36,37,43]. For patients with MI, the average costs in the first year ranged from €3,390 to €27,038 per patient. The costs accrued in the subsequent years ranged between €1,026 and €2,092 per patient per year. The studies that defined which direct costs were covered only took hospitalisation costs into account, ergo no rehabilitation or other costs were covered. Three papers also included indirect costs, though only one accounted for productivity losses [1], as the other two included only overheads [15,17].

### Stroke

The majority of papers reported cost estimates for stroke events: 61 cost estimates in 22 studies were identified (Table 4). The year of costing ranged from 1999 to 2015. Four papers reported on the different severities of stroke [16,28,38,42], and four papers mentioned the type of stroke studied: four reported on ischaemic strokes specifically [16,20,38,47], while one reported haemorrhagic strokes in addition [38]. Four studies reported cost estimates for the first year separated over two periods of six months[18,26,37,42]. One paper reported cost estimates per 3-month cycles [38], and another for just the first six months [13]. Two studies showed that the majority of the cost estimates for stroke events were made up by hospital stay [15,20]. When rehabilitation was considered, it made up an even larger share than hospital stay [18,21]. Three studies also included indirect costs [1,15,18]. Although Tholen *et al*. did take indirect costs into account, the cost estimate in Table 4 does not include productivity losses, since the study reported them separately [16].

### Transient ischaemic attack

Four different costs for TIA were reported in three different studies, with the lowest cost estimate being €587, and the highest amounting to €2,470, reflecting inpatient and outpatient costs, respectively [20]. Hospital stay accounted for the largest share of the costs; €1,748 of the €2,470 were for inpatient stay [20].

### Heart failure

A total of fifteen cost estimates for heart failure were reported in eight studies (Table 6).

The costs for heart failure varied between €945 and €16,561 per patient per year. One study reported a cost estimate for fatal congestive heart failure (HF), which was assumed to be 50% of non-fatal congestive HF events [1]. Three papers reported estimates for the subsequent years of congestive HF [1,24,29]. These ranged between €325 and €6,672. One study reported separate costs for different severities of HF, whether HF went undetected, and by sex [45]. The main cost driver for heart failure is hospital stay, both for the first year as well as the follow-up years, where rehospitalisation accounted for 73% of the costs [44].

### Renal failure

A total of 24 cost estimates associated with renal failure were reported in seven studies (Table 7). Several different types of dialysis were covered in five papers [14,24,29,32,34], reporting fifteen cost estimates in total, which ranged between €54,067 and €89,447. The cost estimates for dialysis in subsequent years were reported to be equal to the estimates for the first year. Three studies reported five cost estimates on renal transplantation [24,29,32]. First year cost estimates ranged between €14,387 and €91,503, and costs for subsequent years were estimated at €2,438 and €3,680. Four cost estimates for end-stage renal disease (ESRD) were reported in three studies [1,32,41]. These costs ranged between €3,640 and €69,440. Adarkwah *et al*. calculated a weighed mean for ESRD patients, taking renal transplantation and different types of dialysis into account [32]. The costs for one year of ESRD were estimated at €42,219, while one year of dialysis and renal transplantation cost €79,112 and €14,387, respectively.

### Revascularisation

A total of seventeen cost estimates for revascularisation were reported in nine studies (Table 8). Two types of revascularisation were assessed; percutaneous coronary intervention (PCI) and coronary arterial bypass grafting (CABG), with respectively ten and seven cost estimates identified. PCI cost estimates ranged from €3,000 to €14,037 [37,40]. For CABG, the cost estimates ranged between €5,441 and €18,010 [33,40].

### Adherence of papers to CHEERS

Table 9 presents the results of the papers that were assessed according to the CHEERS guideline. Overall, papers’ adherence to the checklist was found to be high, even though articles did not explicitly state whether a reporting guideline was used. The items with the lowest amount of adherence were measurement and valuation of preference-based outcomes, assumptions, and characterising heterogeneity. Assumptions were not clearly defined in five studies [13,14,21,35,39], and only partially in two [44,47]. Finally, one study failed to characterise heterogeneity [39].

**Table 9:** Evaluation of the adherence of cost-effectiveness papers to CHEERS.

	Checklist item numbers																		
Authors	1	2	3	4	5	6	7	8	9	13	14	15	16	17	18	19	20	21	22
Adarkwah *et al*. [32]																			
Anastasiadis *et al*. [11]																			
Baeten *et al*. [26]																			
Boersma *et al*. [33]																			
Boersma *et al*. [34]																			
Boyne *et al*. [35]																			
De Vries *et al*. [36]																			
Greving *et al*. [28]																			
Heeg *et al*. [37]																			
Hofmeijer *et al*. [21]																			
Hunt *et al*. [24]																			
Jacobs *et al*. [38]																			
Mazairac *et al*. [14]																			
Nathoe *et al*. [39]																			
Osnabrugge *et al*. [40]																			
Ramos *et al*. [41]																			
Roze *et al*. [29]																			
Stevanović *et al*. [42]																			
Tholen *et al*. [16]																			
Vaidya *et al*. [43]																			
Van Exel *et al*. [13]																			
Van Genugten *et al*. [44]																			
Van Giessen *et al*. [45]																			
Van Haalen *et al*. [1]																			
Vemer *et al*. [46]																			
Verhoef *et al*. [47]																			

*White* yes, *light grey* not applicable *dark grey* partially, *black* no, *CHEERS* Consolidated Health Economic Evaluation Reporting Standards

## Discussion

### Main findings

In this systematic review, we aimed to outline the Dutch cost estimates of six-major T2DM-related clinical events. It was found that many studies reported on cost estimates for MI and stroke, but only a limited number focussed on other T2DM-related clinical events. The most expensive clinical events were found to be related to renal failure, most notably ESRD and dialysis, although some estimates reported stroke and CABG to be a significant source of expenditures as well. MI, TIA, and HF were generally among the least expensive T2DM complications. A large variety in cost estimates was found in the included studies. Adherence to CHEERS guidelines was generally high.

### Interpretation

While some heterogeneity is to be expected when dealing with estimates, most values showed poor agreement, sometimes even between a study and its reference. For example, Verhoef *et al*. reported cost estimates for TIA, but this value was considerably lower than the cost estimates found in their sources [47,61].

Three studies reported cost estimates for stroke separated into minor and major stroke [16,28,38]. In 2018 EUR-corrected values, minor stroke was estimated to cost €9,079, €7,342, and €24,557, respectively, during the first year of stroke and €1,554, €1,256, and €6,177 in subsequent years. Cost estimates for major stroke were €51,779, €41,868, and €58,289 in the first years, and for subsequent years €30,234, €24,447, and €16,475.

As is evident from these values, cost estimates for minor stroke, both in the first year and subsequent years, differed substantially. In contrast, cost estimates for major stroke showed more agreement. A possible reason for these discrepancies could be the definitions of minor and major stroke between the studies. Jacobs *et al*. defined minor stroke as Rankin Scale (mRS) 1–2, with 3–5 being classified as major stroke. While Greving *et al*. and Tholen *et al*. did not specify their definition, it could be that minor stroke was classified as only mRS 1 in these studies, resulting in lower costs as a minor stroke would be less severe. This theory is partly supported by the utilities used in the papers: both in Greving *et al*. and Tholen *et al*., the utility weights for minor stroke were higher than in Jacobs *et al*. However, as Jacobs *et al*. utilise a shorter cycle length, comparing utilities in this manner does not produce a definitive answer.

Stevanović *et al*. separated stroke into three severities: mild, moderate, and severe. Stevanović *et al*. and Jacobs *et al*. both referenced Baeten *et al*. for costs for stroke. However, the former did not utilise mRS to determine severity groups.

While fifteen studies did manage to evaluate resource use and resource costs by means of questionnaires, record files and databases, eleven studies in this review cited sources predating 2005, some even reporting cost estimates in Dutch guilders instead of euros. Estimates derived from older papers may give rise to costs not representative of current costs found in healthcare. Furthermore, this may give rise to a risk of bias, as previous research could be unable to meet the specifications needed, whereas costs derived from first-hand sources, e.g. hospital records, are seen as a more accurate reflection.

Although the Dutch guidelines for economic evaluations prefer the societal perspective [6], only five studies actually took this approach. Moreover, one of these papers explicitly stated that even though the societal perspective was used, the indirect costs due to lost productivity losses were not accounted for, because of the advanced age of the patient group [44]. This means that even though the cost estimate was derived using a societal perspective, it is in fact an incomplete value, considering travel costs for patients and caregivers are accrued regardless of patient age. Regarding the other papers, either a hospital perspective or a healthcare perspective was used. These perspectives lack direct non-medical costs, such as travel costs, as well as indirect non-healthcare costs. Therefore, these cost estimates lack societal costs such as productivity losses.

Tan *et al*. was the only paper included in this review that compared different costing methodologies [15]. In their paper, bottom-up microcosting, top-down microcosting, and gross costing were compared, with gross costing differing the most. If other costing studies had specified their costing methods, it could provide insight into the reason for the heterogeneity found in cost estimates.

In a systematic review about the costs of treating cardiovascular events in Germany, Schmid stated that 80–85% of costs in the first year after MI are spent in the first six months, meaning the other six months of the first year make up for just 15–20% [74]. For the Netherlands, one paper reported first year cost estimates separated in two periods of six months [37]. They found that the cost estimates for the first six months were €10,250 and €2,500 for the subsequent six months. This means that around 80% of the costs in the first year after MI are incurred during the first six months in the Netherlands, similar to Germany. Comparable findings were found for stroke events. In the same paper, Schmid reported that in Germany, 80% of the costs for stroke during the first year were reached within the first half-year. Three papers reported cost estimates for stroke in the Netherlands in periods of six months [18,26,37]. These studies show that between 69 and 74% of the costs during the first year are incurred in the first half-year. From these results, it is clear that the larger part of the first-year costs associated with MI and stroke are incurred within six months of the event. This is to be expected, considering hospitalisation and rehabilitation are the main cost drivers, and are mainly present immediately after an event.

The fact that only three studies reported on the costs associated with TIA illustrates the systematic underreporting of TIA. It being a transient event, a TIA can go unnoticed, even though the patient is at a higher risk of other cardiovascular events, or it is grouped with strokes because of its definition, resulting in an increased number stroke reports, but also decreasing the average reported costs for stroke [75].

A possible cause for the difference in costs found between studies could be the developments in healthcare. For example, more efficient or less expensive procedures could have become the standard. For us, this means that adjusting for inflation would not be enough to carefully compare costs from 2005 to costs from 2015.

To provide context of the studies in which the costs were used, we also reviewed each study using the CHEERS checklist. While the majority of CHEERS items focus on more methodological issues, some items of CHEERS were particularly relevant for this study. These items were “Estimating resources and costs” and “Currency, price date, and conversion”. Generally, the first item was well-addressed, while in the latter, some room for improvement was noted, as either the price date or the conversion method was not mentioned [11,13,16]. However, as the CHEERS statement was developed as a guideline for the reporting of health economic evaluations, the quality of cost estimation cannot be adequately assessed solely with this checklist.

### Strengths and limitations

To the best of our knowledge, this is the first systematic review specifically focussing on Dutch costs of six major T2DM complications. Results can be of relevance for future cost-effectiveness analyses of new type 2 diabetes treatments in the Netherlands. However, also certain limitations have to be acknowledged. Due to our strict inclusion criteria and focus on major cardiovascular and renal complications, no attention could be paid to other T2DM-related events, such as unstable angina, peripheral artery disease, neuropathy, diabetic foot, and retinopathy. Furthermore, the costs associated with micro- and macroalbuminuria were not explicitly reported, although these cannot be considered as events, but rather as bio-factors or risk factors for renal events, and if relevant, these were included in the costs for ESRD. Other T2DM complications are recommended to be included in more comprehensive future studies.

Finally, as most cost estimates reported in the identified studies were based on models or costs derived from guidelines, instead of trial-based values, the generalisability is limited. This means that variance and representativeness of patient samples that make up cost estimates could not be evaluated. Therefore, our review focussed on a descriptive analysis of our findings.

## Conclusions

This systematic review showed that there is substantial variation in reported cost estimates for six major complications associated with T2DM. Most of the studies reported on MI and stroke. Due to a limited amount of papers covering heart failure, revascularization, TIA and renal failure, cost estimates varied widely and transparency regarding cost sources was generally poor. The costing of clinical events related to T2DM should be improved and preferably standardised, if accurate and consistent results in economic models are desired.

## Supporting information

S1 Table PRISMA 2009 checklist. (DOCX)

## References

[1] van Haalen HGM, Pompen M, Bergenheim K, McEwan P, Townsend R, Roudaut M. Cost effectiveness of adding dapagliflozin to insulin for the treatment of type 2 diabetes mellitus in the Netherlands. Clin Drug Investig [Internet]. 2014;34:135–46. Available from: http://www.ncbi.nlm.nih.gov/pubmed/24243529 10.1007/s40261-013-0155-0 24243529

[2] The Emerging Risk Factors Collaboration. Diabetes mellitus, fasting blood glucose concentration, and risk of vascular disease: a collaborative meta-analysis of 102 prospective studies. Lancet [Internet]. Elsevier Ltd; 2010;375:2215–22. Available from: 10.1016/S0140-6736(10)60484-9 20609967 PMC2904878

[3] Statistics of Diabetes mellitus [Internet]. [cited 2016 Dec 12]. Available from: https://www.volksgezondheidenzorg.info/onderwerp/diabetes-mellitus/cijfers-context/huidige-situatie#bron—node-huisartsenregistratie-van-diabetes

[4] CBS StatLine—Population; sex, age, origin and generation, 1 January [Internet]. [cited 2016 Dec 12]. Available from: http://statline.cbs.nl/Statweb/publication/?DM=SLEN&PA=37325eng&D1=0&D2=0&D3=0&D4=0&D5=0&D6=13-20&LA=EN&VW=T

[5] Baan CA, van Baal PHM, Jacobs-van der Bruggen MAM, Verkley H, Poos MJJC, Hoogenveen RT, et al [Diabetes mellitus in the Netherlands: estimate of the current disease burden and prognosis for 2025]. Ned Tijdschr Geneeskd [Internet]. 2009;153:A580 Available from: http://www.ncbi.nlm.nih.gov/pubmed/19785785 19785785

[6] Guideline for economic evaluations in healthcare. Diemen: The National Health Care Institute (Zorginstituut Nederland); 2016. p. 38.

[7] Hakkaart-Van Roijen L, Van der Lindern N, Bouwmans C, Kanters T, Tan SS. Dutch Manual for Costing. Diemen; 10.1371/journal.pone.0187477 PMC5679627 29121647

[8] Moher D, Liberati A, Tetzlaff J, Altman DG, PRISMA Group. Preferred reporting items for systematic reviews and meta-analyses: the PRISMA statement. Ann Intern Med [Internet]. 2009;151:264–9, W64. Available from: http://www.ncbi.nlm.nih.gov/pubmed/21603045 10.7326/0003-4819-151-4-200908180-00135 21603045 PMC3090117

[9] CBS StatLine—Consumer prices; price index 2015 = 100 [Internet]. [cited 2019 Apr 20]. Available from: https://statline.cbs.nl/Statweb/publication/?DM=SLEN&PA=83131eng&D1=0-1&D2=0&D3=12,25,38,51,64,77,90,103,116,129,142,155,168,181,194,207,220,233,246,259,272,285,298,l&LA=EN&HDR=T&STB=G1,G2&VW=T

[10] Husereau D, Drummond M, Petrou S, Carswell C, Moher D, Greenberg D, et al Consolidated health economic evaluation reporting standards (CHEERS)-explanation and elaboration: A report of the ISPOR health economic evaluation publication guidelines good reporting practices task force. Value Heal [Internet]. Elsevier; 2013;16:231–50. Available from: 10.1016/j.jval.2013.02.002 23538175

[11] Anastasiadis K, Fragoulakis V, Antonitsis P, Maniadakis N. Coronary artery bypass grafting with minimal versus conventional extracorporeal circulation; An economic analysis. Int J Cardiol [Internet]. Elsevier Ireland Ltd; 2013;168:5336–43. Available from: 10.1016/j.ijcard.2013.08.006 23992927

[12] Heyde GS, Koch KT, De Winter RJ, Dijkgraaf MGW, Klees MI, Dijksman LM, et al Randomized trial comparing same-day discharge with overnight hospital stay after percutaneous coronary intervention: Results of the Elective PCI in Outpatient Study (EPOS). Circulation. 2007;115:2299–306. 10.1161/CIRCULATIONAHA.105.591495 17420341

[13] van Exel NJA, Koopmanschap MA, Scholte op Reimer W, Niessen LW, Huijsman R. Cost-effectiveness of integrated stroke services. QJM—Mon J Assoc Physicians. 2005;98:415–25. 10.1093/qjmed/hci065 15879443

[14] Mazairac AHA, Blankestijn PJ, Grooteman MPC, Lars Penne E, Van Der Weerd NC, Den Hoedt CH, et al The cost-utility of haemodiafiltration versus haemodialysis in the convective transport study. Nephrol Dial Transplant. 2013;28:1865–73. 10.1093/ndt/gft045 23766337

[15] Tan SS, Rutten FFH, Van Ineveld BM, Redekop WK, Hakkaart-Van Roijen L. Comparing methodologies for the cost estimation of hospital services. Eur J Heal Econ. 2009;10:39–45. 10.1007/s10198-008-0101-x 18340472

[16] Tholen ATR, de Monyé C, Genders TSS, Buskens E, Dippel DWJ, van der Lugt A, et al Suspected carotid artery stenosis: cost-effectiveness of CT angiography in work-up of patients with recent TIA or minor ischemic stroke. Radiology [Internet]. 2010;256:585–97. Available from: http://www.ncbi.nlm.nih.gov/pubmed/20656842 10.1148/radiol.10091157 20656842

[17] Tiemann O. Variations in hospitalisation costs for acute myocardial infarction—a comparison across Europe. Health Econ [Internet]. 2008;17:S33–45. Available from: http://www.ncbi.nlm.nih.gov/pubmed/18186036 10.1002/hec.1322 18186036

[18] van Eeden M, van Heugten C, van Mastrigt GAPG, van Mierlo M, Visser-Meily JMA, Evers SMAA. The burden of stroke in the Netherlands: estimating quality of life and costs for 1 year poststroke. BMJ Open [Internet]. 2015;5:e008220 Available from: http://www.pubmedcentral.nih.gov/articlerender.fcgi?artid=4663410&tool=pmcentrez&rendertype=abstract 10.1136/bmjopen-2015-008220 26614618 PMC4663410

[19] Y van Mastrigt GA, Heijmans J, Severens JL, Fransen EJ, Roekaerts P, Voss G, et al Short-stay intensive care after coronary artery bypass surgery: randomized clinical trial on safety and cost-effectiveness. Crit Care Med. 2006;34:65–75. 10.1097/01.ccm.0000191266.72652.fa 16374158

[20] Buisman LR, Tan SS, Nederkoorn PJ, Koudstaal PJ, Redekop WK. Hospital costs of ischemic stroke and TIA in the Netherlands. Neurology [Internet]. 2015;84:2208–15. Available from: http://www.embase.com/search/results?subaction=viewrecord&from=export&id=L71673323%5Cn 10.1016/j.jval.2014.08.1416 10.1016/j.jval.2014.08.1416 25934858 PMC4456655

[21] Hofmeijer J, Van Der Worp HB, Kappelle LJ, Eshuis S, Algra A, Greving JP. Cost-effectiveness of surgical decompression for space- occupying hemispheric infarction. Stroke. 2013;44:2923–5. 10.1161/STROKEAHA.113.002445 23943217

[22] Peltola M, Quentin W. Diagnosis-related groups for stroke in europe: Patient classification and hospital reimbursement in 11 countries. Cerebrovasc Dis. 2013;35:113–23. 10.1159/000346092 23406838

[23] Soekhlal RR, Burgers LT, Redekop WK, Tan SS. Treatment costs of acute myocardial infarction in the Netherlands. Neth Heart J [Internet]. 2013;21:230–5. Available from: http://www.ncbi.nlm.nih.gov/pubmed/23456884 10.1007/s12471-013-0386-y 23456884 PMC3636331

[24] Hunt B, Glah D, van der Vliet M. Modeling the Long-Term Cost-Effectiveness of IDegLira in Patients with Type 2 Diabetes Who are Failing To Meet Glycemic Targets on Basal Insulin Alone in The Netherlands. Diabetes Ther. Springer Healthcare; 2017;8:753–65. 10.1007/s13300-017-0266-3 28523483 PMC5544604

[25] Oostenbrink JB, Rutten FFH. Cost assessment and price setting of inpatient care in the Netherlands. The DBC case-mix system. Health Care Manag Sci. 2006;9:287–94. 17016935 10.1007/s10729-006-9096-y

[26] Baeten S a, van Exel NJ a, Dirks M, Koopmanschap M a, Dippel DW, Niessen LW. Lifetime health effects and medical costs of integrated stroke services—a non-randomized controlled cluster-trial based life table approach. Cost Eff Resour Alloc [Internet]. 2010;8:21 Available from: http://www.resource-allocation.com/content/8/1/21 10.1186/1478-7547-8-21 21083901 PMC2998455

[27] Struijs JN, van Genugten MLL, Evers SM a a, Ament AJH, Baan C a, van den Bos G a M. Future costs of stroke in the Netherlands: the impact of stroke services. Int J Technol Assess Health Care [Internet]. 2006;22:518–24. Available from: http://www.ncbi.nlm.nih.gov/pubmed/16984687 10.1017/S0266462306051464 16984687

[28] Greving JP, Visseren FLJ, de Wit GA, Algra A. Statin treatment for primary prevention of vascular disease: whom to treat? Cost-effectiveness analysis. BMJ [Internet]. 2011;342:d1672 Available from: http://www.ncbi.nlm.nih.gov/pubmed/21450800 10.1136/bmj.d1672 21450800

[29] Roze S, Duteil E, Smith-Palmer J, de Portu S, Valentine W, de Brouwer BFE, et al Cost-effectiveness of continuous subcutaneous insulin infusion in people with type 2 diabetes in the Netherlands. J Med Econ [Internet]. 2016;19:742–9. Available from: 10.3111/13696998.2016.1167695 26985982

[30] Kauf TL, Velazquez EJ, Crosslin DR, Weaver WD, Diaz R, Granger CB, et al The cost of acute myocardial infarction in the new millennium: Evidence from a multinational registry. Am Heart J. 2006;151:206–12. 10.1016/j.ahj.2005.02.028 16368320

[31] Euro to U.S. dollar annual exchange rate 1999–2016 [Internet]. Available from: https://www.statista.com/statistics/412794/euro-to-u-s-dollar-annual-average-exchange-rate/

[32] Adarkwah CC, Gandjour A, Akkerman M, Evers SM. Cost-effectiveness of Angiotensin-converting enzyme inhibitors for the prevention of diabetic nephropathy in The Netherlands—A Markov model. PLoS One. 2011;6:1–10. 10.1371/journal.pone.0026139 PMC3191181 22022539

[33] Boersma C, Radeva JI, Koopmanschap MA, Voors AA, Postma MJ. Economic evaluation of valsartan in patients with chronic heart failure: Results from Val-HeFT adapted to The Netherlands. J Med Econ. 2006;9:121–31.

[34] Boersma C, Gansevoort RT, Pechlivanoglou P, Visser ST, van Toly FFJ, de Jong-van den Berg LTW, et al Screen-and-treat strategies for albuminuria to prevent cardiovascular and renal disease: Cost-effectiveness of nationwide and targeted interventions based on analysis of cohort data from the Netherlands. Clin Ther [Internet]. Excerpta Medica Inc.; 2010;32:1103–21. Available from: 10.1016/j.clinthera.2010.06.013 20637965

[35] Boyne JJJ, Van Asselt ADI, Gorgels APM, Steuten LMG, De Weerd G, Kragten J, et al Cost-effectiveness analysis of telemonitoring versus usual care in patients with heart failure: the TEHAF-study. J Telemed Telecare [Internet]. 2013;19:242–8. Available from: http://www.ncbi.nlm.nih.gov/pubmed/24163233 10.1177/1357633X13495478 24163233

[36] De Vries FM, Denig P, Visser ST, Hak E, Postma MJ. Cost-effectiveness of statins for primary prevention in patients newly diagnosed with type 2 diabetes in the Netherlands. Value Heal [Internet]. Elsevier; 2014;17:223–30. Available from: 10.1016/j.jval.2013.12.010 24636380

[37] Heeg BMS, Peters RJG, Botteman M, van Hout BA. Long-term clopidogrel therapy in patients receiving percutaneous coronary intervention. Pharmacoeconomics [Internet]. 2007;25:769–82. Available from: https://ideas.repec.org/cgi-bin/htsearch?cmd=Search!&db=01/01/1990&de=&dt=range&fmt=long&m=all&np=9&ps=50&q=(saving++%7C+micro-saving+%7Cmicrosaving+%7C+Aflatoun+%7C+YouthSafe+)+++(“randomized+control*+trial”+%7C++“randomised+contr 10.2165/00019053-200725090-00005 17803335

[38] Jacobs MS, Kaasenbrood F, Postma MJ, van Hulst M, Tieleman RG. Cost-effectiveness of screening for atrial fibrillation in primary care with a handheld, single-lead electrocardiogram device in the Netherlands. Europace [Internet]. 2018;20:12–8. Available from: http://www.ncbi.nlm.nih.gov/pubmed/27733465 10.1093/europace/euw285 27733465

[39] Nathoe HM, Dijk D Van, Jansen EWL, Borst C, Grobbee DE, Jaegere PPT D. Off-pump coronary artery bypass surgery compared with stent implantation and on-pump bypass surgery: clinical outcome and cost-effectiveness at one year. Heart. 2005;13:259–68. PMC2497251 25696506

[40] Osnabrugge RL, Magnuson E a., Serruys PW, Campos CM, Wang K, van Klaveren D, et al Cost-effectiveness of percutaneous coronary intervention versus bypass surgery from a Dutch perspective. Heart [Internet]. 2015;1–9. Available from: 10.1136/heartjnl-2015-307821 26552756

[41] Ramos IC, Versteegh MM, de Boer RA, Koenders JMA, Linssen GCM, Meeder JG, et al Cost Effectiveness of the Angiotensin Receptor Neprilysin Inhibitor Sacubitril/Valsartan for Patients with Chronic Heart Failure and Reduced Ejection Fraction in the Netherlands: A Country Adaptation Analysis Under the Former and Current Dutch Pharmacoeco. Value Health [Internet]. Elsevier Inc.; 2017;20:1260–9. Available from: 10.1016/j.jval.2017.05.013 29241885

[42] Stevanović J, Pompen M, Le HH, Rozenbaum MH, Tieleman RG, Postma MJ. Economic evaluation of apixaban for the prevention of stroke in non-valvular atrial fibrillation in the Netherlands. PLoS One. 2014;9. 10.1371/journal.pone.0103974 PMC4122386 25093723

[43] Vaidya A, Severens JL, Bongaerts BWC, Cleutjens KBJM, Nelemans PJ, Hofstra L, et al High-sensitive troponin T assay for the diagnosis of acute myocardial infarction: an economic evaluation. BMC Cardiovasc Disord [Internet]. 2014;14:77 Available from: 10.1186/1471-2261-14-77 24927776 PMC4065542

[44] van Genugten MLL, Weintraub WS, Zhang Z, Voors AA. Cost-effectiveness of eplerenone plus standard treatment compared with standard treatment in patients with myocardial infarction complicated by left ventricular systolic dysfunction and heart failure in the Netherlands. Neth Heart J [Internet]. 2005;13:393–400. Available from: http://ovidsp.ovid.com/ovidweb.cgi?T=JS&CSC=Y&NEWS=N&PAGE=fulltext&D=emed7&AN=2005519346%5Cn http://nhs5531173.on.worldcat.org/atoztitles/link?sid=OVID:embase&id=pmid:&id=doi:&issn=0929-7456&isbn=&volume=13&issue=11&spage=393&pages=393-400&date=2005&title= 25696430 PMC2497358

[45] van Giessen A, Boonman-de Winter LJM, Rutten FH, Cramer MJ, Landman MJ, Liem AH, et al Cost-effectiveness of screening strategies to detect heart failure in patients with type 2 diabetes. Cardiovasc Diabetol [Internet]. BioMed Central; 2016;15:48 Available from: http://www.cardiab.com/content/15/1/48 10.1186/s12933-016-0363-z 27001409 PMC4802923

[46] Vemer P, Rutten-van Mölken MPMH. Crossing borders: factors affecting differences in cost-effectiveness of smoking cessation interventions between European countries. Value Health [Internet]. 2010;13:230–41. Available from: 10.1111/j.1524-4733.2009.00612.x 19804435

[47] Verhoef TI, Redekop WK, Hasrat F, de Boer A, Maitland-van der Zee AH. Cost Effectiveness of New Oral Anticoagulants for Stroke Prevention in Patients with Atrial Fibrillation in Two Different European Healthcare Settings. Am J Cardiovasc Drugs. 2014;14:451–62. 10.1007/s40256-014-0092-1 25326294 PMC4250561

[48] Redekop WK, Koopmanschap MA, Rutten GEHM, Wolffenbuttel BHR, Stolk RP, Niessen LW. Resource consumption and costs in Dutch patients with type 2 diabetes mellitus. Results from 29 general practices. Diabet Med [Internet]. 2002;19:246–53. Available from: http://www.ncbi.nlm.nih.gov/pubmed/11918627 11918627 10.1046/j.1464-5491.2002.00654.x

[49] Voss GBWE, Hasman A, Rutten F, de Zwaan C, Carpay JJ. Explaining cost variations in DRGs “Acute Myocardial Infarction” by severity of illness. Health Policy (New York). 1994;28:37–50. 10.1016/0168-8510(94)90019-1 10134586

[50] Thurston SJ, Heeg B, de Charro F, van Hout B. Cost-effectiveness of clopidogrel in STEMI patients in the Netherlands: a model based on the CLARITY trial. Curr Med Res Opin [Internet]. 2010;26:641–51. Available from: http://www.ncbi.nlm.nih.gov/pubmed/20070142 10.1185/03007990903529267 20070142

[51] Isaaz K, Coudrot M, Sabry MH, Cerisier A, Lamaud M, Robin C, et al Return to work after acute ST-segment elevation myocardial infarction in the modern era of reperfusion by direct percutaneous coronary intervention. Arch Cardiovasc Dis [Internet]. Elsevier Masson SAS; 2010;103:310–6. Available from: 10.1016/j.acvd.2010.04.007 20619241

[52] de Boer MJ, van Hout BA, Liem AL, Suryapranata H, Hoorntje JC, Zijlstra F. A cost-effective analysis of primary coronary angioplasty versus thrombolysis for acute myocardial infarction. Am J Cardiol [Internet]. 1995;76:830–3. Available from: http://www.ncbi.nlm.nih.gov/pubmed/7572666 10.1016/s0002-9149(99)80238-0 7572666

[53] Johannesson M, Jönsson B, Kjekshus J, Olsson AG, Pedersen TR, Wedel H. Cost effectiveness of simvastatin treatment to lower cholesterol levels in patients with coronary heart disease. Scandinavian Simvastatin Survival Study Group. N Engl J Med [Internet]. 1997;336:332–6. Available from: http://www.nejm.org/doi/abs/10.1056/NEJM199701303360503 10.1056/NEJM199701303360503 9011785

[54] Serruys PW, Van HB, Bonnier H, Legrand V, Garcia E, Macaya C, et al Randomised comparison of implantation of heparin-coated stents with balloon angioplasty in selected patients with coronary artery disease (Benestent II)[erratum appears in Lancet 1998 Oct 31;352(9138):1478]. Lancet [Internet]. 1998;352:673–81. Available from: http://www.sciencedirect.com/science/article/pii/S014067369711128X%5Cnhttp://ac.els-cdn.com/S014067369711128X/1-s2.0-S014067369711128X-main.pdf?_tid=f723f150-350b-11e3-8dcc-00000aab0f01&acdnat=1381781305_7c22664c05bdbd830bbfc4896b338034 10.1016/s0140-6736(97)11128-x 9728982

[55] Bergman L, van der Meulen JH, Limburg M, Habbema JD. Costs of medical care after first-ever stroke in The Netherlands. Stroke [Internet]. 1995;26:1830–6. Available from: http://www.ncbi.nlm.nih.gov/pubmed/7570734 10.1161/01.str.26.10.1830 7570734

[56] Van Hout BA, Simoons ML. Cost-effectiveness of HMG coenzyme reductase inhibitors: Whom to treat? Eur Heart J. 2001;22:751–61. 10.1053/euhj.2000.2308 11350107

[57] Lindgren P, Glader E-L, Jönsson B. Utility loss and indirect costs after stroke in Sweden. Eur J Cardiovasc Prev Rehabil [Internet]. 2008;15:230–3. Available from: 10.1097/HJR.0b013e3282f37a22 18391653

[58] Buskens E, Nederkoorn PJ, Buijs-Van Der Woude T, Mali WPTM, Kappelle LJ, Eikelboom BC, et al Imaging of carotid arteries in symptomatic patients: cost-effectiveness of diagnostic strategies. Radiology [Internet]. 2004;233:101–12. Available from: http://www.ncbi.nlm.nih.gov/pubmed/15333770 10.1148/radiol.2331030863 15333770

[59] Niessen LW, Dippel DW, Limburg M. [Calculation of costs of stroke, cost effectiveness of stroke units and secondary prevention in patients after a stroke, as recommended by revised CBO practice guideline ‘Stroke’]. Ned Tijdschr Geneeskd [Internet]. 2000;144:1959–64. Available from: http://www.ncbi.nlm.nih.gov/pubmed/11048560 11048560

[60] van Exel J, Koopmanschap M a, van Wijngaarden JD, Scholte op Reimer WJ. Costs of stroke and stroke services: Determinants of patient costs and a comparison of costs of regular care and care organised in stroke services. Cost Eff Resour Alloc. 2003;1:2 10.1186/1478-7547-1-2 12773219 PMC156021

[61] Buisman LR, Tan SS, Koudstaal PJ, Nederkoorn PJ, Redekop WK. Hospital Costs Of Ischemic Stroke And Transient Ischemic Attack In The Netherlands. Value Health [Internet]. 2014;17:A485 Available from: http://www.ncbi.nlm.nih.gov/pubmed/27201429 10.1016/j.jval.2014.08.1416 27201429

[62] Stevanovic J, Denee L, Koenders JM, Postma MJ. Incidence Description and Costs of Acute Heart Failure in the Netherlands. Value Health [Internet]. 2014;17:A328 Available from: http://www.ncbi.nlm.nih.gov/pubmed/27200554 10.1016/j.jval.2014.08.597 27200554

[63] Postmus D, Abdul Pari AA, Jaarsma T, Luttik ML, Van Veldhuisen DJ, Hillege HL, et al A trial-based economic evaluation of 2 nurse-led disease management programs in heart failure. Am Heart J [Internet]. Mosby, Inc.; 2011;162:1096–104. Available from: 10.1016/j.ahj.2011.09.019 22137084

[64] Ericson L, Bergfeldt L, Björholt I. Atrial fibrillation: The cost of illness in Sweden. Eur J Heal Econ. 2011;12:479–87. 10.1007/s10198-010-0261-3 PMC3160555 20593297

[65] Costs of diseases 2007. 2011.

[66] Cavallo MC, Sepe V, Conte F, Abelli M, Ticozzelli E, Bottazzi A, et al Cost-effectiveness of kidney transplantation from DCD in Italy. Transplant Proc [Internet]. 2014;46:3289–96. Available from: http://www.ncbi.nlm.nih.gov/pubmed/25498039 10.1016/j.transproceed.2014.09.146 25498039

[67] Niessen LW, Dijkstra R, Hutubessy R, Rutten GE, Casparie AF. Lifetime health effects and costs of diabetes treatment. Neth J Med. 2003;61:355–64. 14768718

[68] Clarke P, Leal J, Kelman C, Smith M, Colagiuri S. Estimating the cost of complications of diabetes in Australia using administrative health-care data. Value Heal [Internet]. International Society for Pharmacoeconomics and Outcomes Research (ISPOR); 2008;11:199–206. Available from: 10.1111/j.1524-4733.2007.00228.x 18380631

[69] van der Mei SF, Kuiper D, Groothoff JW, van den Heuvel WJA, van Son WJ, Brouwer S. Long-Term Health and Work Outcomes of Renal Transplantation and Patterns of Work Status During the End-Stage Renal Disease Trajectory. J Occup Rehabil [Internet]. 2011;21:325–34. Available from: 10.1007/s10926-011-9317-1 21656250 PMC3173627

[70] de Wit GA, Ramsteijn PG, de Charro FT. Economic evaluation of end stage renal disease treatment. Health Policy (New York) [Internet]. 1998;44:215–32. Available from: http://ovidsp.ovid.com/ovidweb.cgi?T=JS&CSC=Y&NEWS=N&PAGE=fulltext&D=med4&AN=10182294 10.1016/s0168-8510(98)00017-7 10182294

[71] Wijnen E, Planken N, Keuter X, Kooman JP, Tordoir JHM, de Haan MW, et al Impact of a quality improvement programme based on vascular access flow monitoring on costs, access occlusion and access failure. Nephrol Dial Transplant [Internet]. 2006;21:3514–9. Available from: http://www.ncbi.nlm.nih.gov/pubmed/16921189 10.1093/ndt/gfl424 16921189

[72] Manns B, Tonelli M, Yilmaz S, Lee H, Laupland K, Klarenbach S, et al Establishment and maintenance of vascular access in incident hemodialysis patients: a prospective cost analysis. J Am Soc Nephrol [Internet]. 2005;16:201–9. Available from: 10.1681/ASN.2004050355 15563567

[73] van Os N, Niessen LW, Bilo HJ, Casparie a F, van Hout B a. Diabetes nephropathy in the Netherlands: a cost effectiveness analysis of national clinical guidelines. Health Policy [Internet]. 2000;51:135–47. Available from: http://www.ncbi.nlm.nih.gov/pubmed/10720684 10720684 10.1016/s0168-8510(00)00063-4

[74] Schmid T. Costs of treating cardiovascular events in Germany: a systematic literature review. Health Econ Rev [Internet]. Health Economics Review; 2015;5:27 Available from: http://www.pubmedcentral.nih.gov/articlerender.fcgi?artid=4580672&tool=pmcentrez&rendertype=abstract 10.1186/s13561-015-0063-5 26400849 PMC4580672

[75] Simmons BB, Cirignano B, Gadegbeku AB. Transient ischemic attack: Part I. Diagnosis and evaluation. Am Fam Physician. 2012;86:521–6. 23062043

